# Translation and validation of the vertigo symptom scale into German: A cultural adaption to a wider German-speaking population

**DOI:** 10.1186/1472-6815-12-7

**Published:** 2012-07-02

**Authors:** Thomas Gloor-Juzi, Annette Kurre, Dominik Straumann, Eling D de Bruin

**Affiliations:** 1 Physiotherapy Occupational Therapy, University Hospital Zurich, Zurich, Switzerland; 2 Interdisciplinary Centre for Vertigo & Balance Disorders, Departments of ENT, Neurology & Psychiatry, University Hospital Zurich, Zurich, Switzerland; 3 Institute of Human Movement Sciences and Sport, ETH Zurich, Zurich, Switzerland

## Abstract

**Background:**

Dizziness and comorbid anxiety may cause severe disability of patients with vestibulopathy, but can be addressed effectively with rehabilitation. For an individually adapted treatment, a structured assessment is needed. The Vertigo Symptom Scale (VSS) with two subscales assessing vertigo symptoms (VSS-VER) and associated symptoms (VSS-AA) might be used for this purpose. As there was no validated VSS available in German, the aim of the study was the translation and cross-cultural adaptation in German (VSS-G) and the investigation of its reliability, internal and external validity.

**Methods:**

The VSS was translated into German according to recognized guidelines. Psychometric properties were tested on 52 healthy controls and 202 participants with vestibulopathy. Internal validity and reliability were investigated with factor analysis, Cronbach’s α and ICC estimations. Discriminant validity was analysed with the Mann–Whitney-*U*-Test between patients and controls and the ROC-Curve. Convergent validity was estimated with the correlation with the Hospital Anxiety Subscale (HADS-A), Dizziness Handicap Inventory (DHI) and frequency of dizziness.

**Results:**

Internal validity: factor analysis confirmed the structure of two subscales. Reliability: VSS-G: α = 0.904 and ICC (CI) =0.926 (0.826, 0.965). Discriminant validity: VSS-VER differentiate patients and controls ROC (CI) =0.99 (0.98, 1.00). Convergent validity: VSS-G correlates with DHI (r = 0.554) and frequency (T = 0.317). HADS-A correlates with VSS-AA (r = 0.452) but not with VSS-VER (r = 0.186).

**Conclusions:**

The VSS-G showed satisfactory psychometric properties to assess the severity of vertigo or vertigo-related symptoms. The VSS-VER can differentiate between healthy subjects and patients with vestibular disorders. The VSS-AA showed some screening properties with high sensitivity for patients with abnormal anxiety.

## Background

With a lifetime prevalence of 29.3 percent in the general German adult population, moderate to severe vertigo or dizziness is a frequent and often recurrent symptom [1]. Furthermore, as 80 percent of the patients reported severe limitations in daily activities [1], a considerable curtailing in health-related quality of life may be expected. Moderate to severe vertigo or dizziness can interrelate with psychiatric disorders, especially anxiety [2-6], which may increase disability [4].

The severity of symptoms and the perceived limitations in activities of daily life can be addressed effectively by individually tailored vestibular rehabilitation based on vestibular habituation to movement [7-10], optionally combined with cognitive behavioural therapy [11-13]. A requirement for an individually tailored treatment is an assessment, which has to be carried out as thoroughly as possible. In order to focus on the symptoms associated with dizziness, the Vertigo Symptom Scale (VSS) was considered to be a key instrument. The VSS consists of two subscales: 1) the Vertigo scale (VSS-VER) which assesses symptoms mainly associated with disorders of the vestibular system and 2) the Anxiety and Autonomic symptom scale (VSS-AA) for the assessment of a group of generic symptoms which may be associated with autonomic arousal or somatic expressions of anxiety [14]. The original VSS as well as a Spanish and a Swedish version showed acceptable psychometric properties [5,15]. However, at the beginning of the study (Spring 2007) there was no validated VSS available in German. The author of the original VSS, L. Yardley [14] was contacted and she confirmed that she had no knowledge of a currently on-going validation. Hence, she approved the German translation and cross-culturally adaptation of the VSS and its validation. Thereby we intended to apply an approved methodology guideline for the translation process [16,17] and to take in consideration existing cultural and language differences between the German-speaking populations in Europe (e.g., Austria, Germany, Belgium, Switzerland, Italy) [18]. A German version of the VSS from Tschan et al. [19] that in the meantime was published did not specifically comply with guidelines (e.g. use informed and uninformed translators and back-translators) and neither considered the possibility of cross-cultural differences, which might have biased their results. For this reason the primary aim of the study was the translation and the cross-cultural adaptation of the VSS into German for the German-speaking population of Switzerland and to investigate its internal validity and reliability for patients with vestibulopathy. The secondary aim of the study was the investigation of the discriminant and convergent validity as parts of external validity determination.

## Methods

### Translation and cross cultural adaptation of the VSS

The VSS is a self-completed, 34-item questionnaire (19 items for VSS-VER and 15 for VSS-AA). The frequency of the symptoms is rated on a Likert-scale: 0 points: “never”, 1 point: “a few times (1–3 times a year)”, 2 points: “several times (4–12 times a year)”, 3 points: “quite often (on average, more than once a month)” and 4 points: “very often (on average more than once a week)”. The total score aims to figure out the latent dimension of severity of dizziness and ranges from 0 points (no symptoms) to 136 points (severe vertigo).

The translation and cross-cultural adaptation into German was performed in six steps, according to the international guidelines for self-reported measures published by the American Association of Orthopaedic Surgeons Outcome Committee [16] and additional information about requirements for translators from Wild et al. [17]. First step: two independent German VSS-translations were requested in order to reflect possible ambiguous wording. Two native German-speaking translators with excellent English language skills translated independently the VSS into German. Moreover, as recommended by Wild [17], one translator was aware of the measured concept of the VSS (health professional), the other not. Second step: meeting of both of the translators and an observer to keep record of the merger of both translations and in order to resolve discrepancies. Third step: back translation of the previously obtained German version into English by two independent native English speaking translators with German language skills, both translators were withheld of the original version of the VSS, again one person was aware of the concept the other not. This step was necessary in order to bring out unclear wording or cultural peculiarities in symptom description and to help to assure a consistent translation of the content of each item. Fourth step: an expert committee consisting of the four translators, the methodologist and two health and language professionals produced the pre-final version of the VSS. Prior to this meeting; Lucy Yardley (author of the original VSS) compared the original VSS with both back translations. Her comments were also considered in the discussion. Fifth step: the pre-final VSS-G was pilot tested according to the recommendations of Wild [17] in a group of fourteen patients who fulfilled the inclusion/exclusion criteria below. Sixth step: The transcription of the patient interviews and the documents of the preceding steps were analysed in the final stage and the final version of the VSS-G was written. Since cross-cultural differences exist between German-speaking countries [18] and both Swiss and German patients frequent our hospital, special consideration was given to specifically include both German and Swiss cultural background translators and physiotherapists in our research team.

### Participants

The study participants were suffering from vertigo, dizziness, or unsteadiness associated with a diagnosed vestibular disorder for at least one month. They had to be between 18 and 75 years old, capable of walking and independently managing approximately 50% of their daily tasks and have good German language skills. The exclusion criteria comprised dizziness or unsteadiness exclusively due to cardiopulmonary diseases, musculoskeletal problems or neurologic disorders like severe paresis, spasticity, cerebellar ataxia, extrapyramidal diseases, or sensory loss. Furthermore, patients with diagnosed dementia, psychiatric disorders or blindness were excluded. Recruitment took place between July 2007 and July 2009 through the Interdisciplinary Centre for Vertigo & Balance Disorders, Departments of ENT, Neurology and Psychiatry at the University Hospital Zurich. All patients who were referred to the department were asked to participate in this study. Those who met the criteria and submitted their signed written informed consent were included in the study. Healthy participants were mainly family members or friends of the authors and their colleagues and were also included after submitting their signed written informed consent. The approval of the ethics committee of the Canton of Zurich was obtained in accordance with the Declaration of Helsinki.

### Measures

The Hospital Anxiety and Depression Scale (HADS) is a 14-item questionnaire measuring psychological distress. It is divided into two subscales assessing non-somatic symptoms of anxiety and depression [20]. Although the whole questionnaire was submitted to the participants, only the results of the anxiety subscale are reported here. The items are rated from 0 to 3 points and the score ranges from 0 (no sign of anxiety) to 21 (maximum level of anxiety). The validity of the HADS has been demonstrated by a large number of patient groups. The psychometric properties of the German version are good and the recommended cut-off score for screening abnormal anxiety is eleven [21].

The Dizziness Handicap Inventory (DHI) [22] is a 25-item questionnaire designed to evaluate the precipitating physical factors associated with dizziness and unsteadiness as well as the functional and emotional consequences of vestibular disease[22]. The items were rated with “yes” (4 points), “sometimes” (2 points) and “no” (0 points). The total score ranges from zero (no disability) to 100 (severe disability). The original version demonstrated good face validity, internal consistency and test-retest reliability in a population with different aetiologies of dizziness and unsteadiness[22]. A valid German version is available [23].

xThree items of the University of California Los Angeles-Dizziness Questionnaire UCLA-DQ [24] were used to rate the overall perceived frequency of dizziness (UCLA-DQ1), its intensity (UCLA-DQ2) and the impact of dizziness on daily activities (UCLA-DQ3). The problems were rated on a scale ranging from 1 (lowest level of the severity of the problem) to 5 (highest level). Furthermore the patients had to rate their level of disability induced by dizziness as no disability (0 points), mild (1), moderate (2) or severe (3 points).

**Figure 1:**
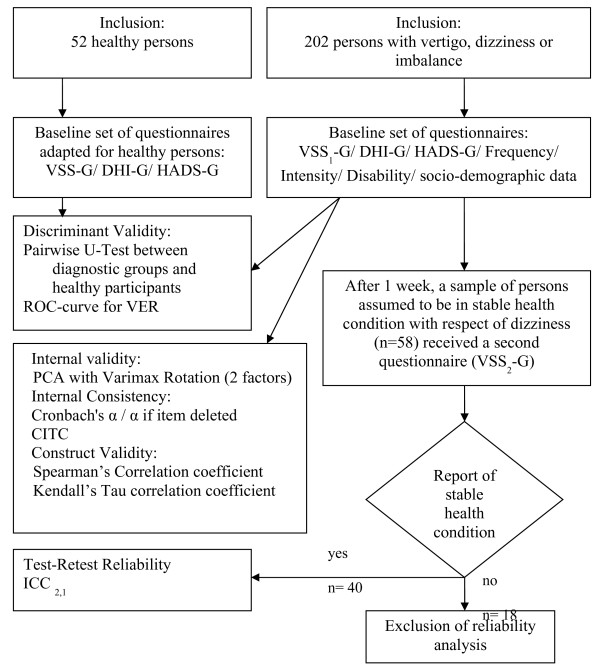
Flow-chart of data collection and analysis procedure.

The postal data collection procedure for the reliability and validity investigation are summarized in Figure 1. The test-retest reliability analysis was carried out on 40 complete pairs of questionnaires [25]. The data collection procedure was controlled on a daily basis. The quality was assured by contacting the patients by phone in order to clear up ambiguous responses, to fill gaps in the questionnaires or to remind them to return the questionnaires. All the analysis was performed on IBM PASW Statistics (formerly SPSS Statistics) version 18 software.

### Internal validity determination

#### Factor analysis

According to the preceding studies [14,19], a principal component analysis (PCA) for two factors with Varimax rotation was performed, in order to compare the loadings of the items on both factors representing the structure of the subscales (VSS-VER and VSS-AA). Prior to the statistical analysis, ceiling or floor effects in the VSS-G or the subscales were checked in the study population.

#### Reliability determination

Cronbach's α coefficient was calculated to investigate the internal consistency of the VSS-G and both subscales. For psychometric scales Cronbach’s α > 0.8 is generally recommended [26]. The Cronbach's α coefficient was also estimated with each item excluded, where the difference between α-total and α-with-the-item-deleted should not be greater than 0.1 [27]. The corrected item-total correlations (CI-TC) were calculated to investigate the strength of relationship between a single item and the other items in each of the scales. CI-TC should range between 0.20 and 0.40 [28], with the minimal recommended value of 0.2 [26]. The intra-tester reliability was calculated with the two-way random model with single measurement intraclass correlation coefficient (ICC_2,1_ absolute agreement type) [29,30].

### External validity determination

#### Discriminant validity

was analysed by comparing the median scores of VSS-G, VSS-VER and VSS-AA between a group of 52 healthy subjects and the three diagnostic subgroups: “1 = peripheral vestibular disorder, 2 = central vestibular disorder and 3 = multifactorial/multisensory disorders”. Significant differences between the groups were investigated with the Mann–Whitney-*U*-Test for independent groups. If any significant difference between the groups was found the ROC curve and the Youden Index [31] were carried out. The Mann–Whitney-*U*-Test was further used to investigate the ability of the VSS-AA median score to differentiate abnormal anxiety as defined by the HADS-A (score ≥ 11).

#### Convergent validity

of the VSS-G and its subscales was analysed using the Spearman’s correlations to investigate the association of VSS-G, VSS-VER and VSS-AA with HADS-A and DHI. Kendall’s Tau rank correlation was performed to analyse the associations of the VSS with the estimated level of disability and the 3 items of the UCLA-DQ: 1) frequency, 2) intensity and 3) impact of dizziness on daily activities.

## Results

After the translation and pilot testing process of a prefinal version, the VSS-G was tested with 202 participants with vertigo or dizziness and 52 healthy subjects (Table 1: characteristics of participants).

**Table 1:** Characteristics of participants

	**Participants with vestibular disorder (n=202)**		**Subgroup for reliability testing (n=40)**		**Healthy participants (n=52)**	
**Age in years: mean (SD)**			50.0	(13.47)	50.7	(13.63)	46.7	(13.12)
**Gender: n women/men (% women):**			124/78	(61.4)	23/17	(57.5)	28/24	(53.8)
**Independence or need of support for ADL/household: n (%)**								
independent			162	(80.2)	33	(82.5)	52	(100.0)
need of occasional support			27	(13.4)	6	(15.0)	0	
need of regular weekly or daily support			13	(6.4)	1	(2.5)	0	
**Duration of dizziness: n (%)**								
dizziness since ≥ 1 month, less than 6 mo			58	(28.7)	8	(20.0)		
dizziness since ≥ 6 mo up to 12 mo			27	(13.4)	7	(17.5)		
dizziness since > 12 mo	117	(57.9)	25	(62.5)				
**Diagnosis: n (%)**								
**Unilateral peripheral vestibular disorder:**			**73**	**(36.14)**	**10**	(**25.00**)		
unilat BPPV			26	(12.9)	3	(7.5)		
unilat. Morbus Meniere			27	(13.4)	4	(10.0)		
unilat. neuritis vestibularis			4	(2.0)	2	(5.0)		
others			16	(7.9)	1	(2.5)		
**Bilateral peripheral vestibular disorder:**			**17**	**(8.4)**	**4**	(**10.0**)		
bilat. BPPV			2	(1.0)	0			
bilat. M. Meniere			1	(.5)	0			
bilat. neuritis vestibularis			4	(2.0)	3	(7.5)		
others			10	(5.0)	1	(2.5)		
**Central vestibular disorder:**			**73**	**(36.1)**	**18**	(**45.0**)		
incomplete central compensation			7	(3.5)	2	(5.0)		
psycho-physical vertigo			17	(8.4)	2	(5.0)		
others (e.g. vest. Migraine)			49	(24.3)	14	(35.0)		
**Multifactorial/multisensory vestibular disorder:**			**39**	**(19.3)**	**8**	(**20.0**)		
**Self percieved level of disability: n (%)**								
little			56	(27.7)	7	(17.5)		
moderate			100	(49.5)	23	(57.5)		
severe			46	(22.8)	10	(25.0)		
**UCLA 1: frequency of dizziness: n (%)**								
rarely			23	(11.4)	4	(10.0)		
sometimes			91	(45.0)	19	(47.5)		
approximately half of the time			39	(19.3)	10	(25.0)		
usually			34	(16.8)	4	(10.0)		
always			15	(7.4)	3	(7.5)		
**UCLA 2: Intensity of dizziness: n (%)**								
very mild			10	(5.0)	2	(5.0)		
mild			23	(11.4)	4	(10.0)		
moderate			78	(38.6)	17	(42.5)		
moderately severe			73	(36.1)	13	(32.5)		
severe			18	(8.9)	4	(10.0)		
**UCLA 3: dizziness induced limitations in activities or participation: n (%)**								
no effect at all			15	(7.4)	3	(7.5)		
continuing all activities but allowance for dizziness			39	(19.3)	8	(20.0)		
continuing most of the activities			79	(39.1)	18	(45.0)		
continuing some of the activities			51	(25.2)	9	(22.5)		
unable to continue any of the activities			18	(8.9)	2	(5.0)		

### Translation and pilot testing of the prefinal translation of the VSS-G

Throughout the translation and cross cultural adaptation process the translators encountered some difficulties to adapt definitions like: unsteadiness, actually fall, light-headed, swimmy, giddy or walk properly for the items of the VSS-VER. There were also some discussion needed to adapt descriptions of symptoms of the VSS-AA like: about to black out, tingling, prickling, spots before the eyes, heart pounding, soreness in muscles, stomach churning, hot or cold spells (not to confound with symptoms of menopause). However the expert committee in the fourth step of the process reached consensus for each item and the patients (in the pilot testing) were able to recognise and associated the described symptoms with their vestibular problems. Compared to the German VSS version that was published during our data gathering process [19], we found differences in the way sentences were constructed. This could be due to cross-cultural differences between the German-speaking populations in Germany and Switzerland [18]. However, the wording used to describe the symptoms was essentially the same.

For the pilot testing (cognitive debriefing) of the pre-final version, 14 patients (8 male) with vestibular disorders (8 peripheral (57%), 5 central vestibular disorder (37%) and 1 multifactorial (7%) causes of dizziness could be included. The mean age (SD) was 60.5 (14.13) years, the mean scores of the DHI (SD) = 46 (20.0), the VSS = 49 (25) and the subscales VSS-VER = 24 (14), VSS-AA = 25 (13). The participants estimated the matching of the contents of the VSS items with their symptoms at 92.2 percent (SD 6.9). One outlier (more than 3 standard deviations differences to the mean), who could not cite any additional problem or symptom the VSS was not accounting for, was excluded from the calculation. Fatigue and weariness were the most cited problems (3 from 14) which are not included in the VSS. However, to the best of our knowledge, even if both symptoms could appear with vestibular disorders, there is no association of fatigue as a symptom of dizziness described in literature. Therefore there was no item added to the VSS-G. The other cited symptoms were diarrhoea, inflammation of the pharynx (sensation of burning while swallowing), polyuria and incontinence, tickling and itching in the ears, hunger and panic. Some patients missed items with vertigo-causing activities (e.g. walking, head movements) or emotions like feeling helpless or alone, fear of becoming dependent or losing work. These propositions underpin the necessity to assess limitations in activity, participation and emotional distress but are beyond the scope of the VSS. Based on these results, the VSS-G can be considered to be complete.

Although most patients declared to easily understand the content of the VSS-G, the interviewers noticed among seven persons (50% of participants) difficulties to understand how to deal with the two-tiered questions 1, 7 and 18. These items were used to determine the frequency range of a vertigo symptom with different duration. The main problem seemed to be the two time-related concepts of symptom duration and frequency. These symptoms might appear or increase with activities (e.g. walking, head movements) and will last through the whole activity, which possibly is an additional confusing time-related aspect. A similar problem was also described by Holmberg for a Swedish version of the VSS [32], in order to counteract this problem we used a clear structure in the layout of these items , even so health professionals may take into account, that patients will need assistance for rating these items. Upon suggestion of some patients, the definitions of the scale-ranking were reported on top margin of each page of the questionnaire (Additional file 1).

### Internal validity determination

#### Principal component analysis

Table 2 contains the results of the two factors extraction with a PCA and Varimax rotation and, for the sake of comparison, the loadings of previous versions and translations of the VSS were also displayed [5,14,19]. Compared to the original VSS, 87% (13/15) of the items could be clearly associated to the VSS-AA scale. Indeed, sixteen items loaded > 0.5 on factor one and thirteen of these items were attributed to Yardley’s original VSS-AA subscale. The three remaining items were vertigo symptoms lasting for less than 2 minutes (1a, 7a, 18a). Their allocation to the VSS-VER subscale will be explained below. Two items (9, 13) showed a clearly higher loading on factor one than on factor two; these loadings were between 0.4 and 0.5. With high face validity, these two items were integrated in the VSS-AA subscale of VSS-G, so that the VSS-AA subscale was identical to the AA subscale of the original VSS.

**Table 2:** PCA with Varimax Rotation: table for two factor loadings comparison

**Item**	**Factor 1**					**Factor 2**				
	**CH**	**Germany**	**Mexic hosp**	**UK hosp.**	**Prim care (UK)**	**Swiss**	**Germany**	**Mexic hosp**	**UK hosp**	**Prim care (UK)**
01. Things spinning/moving (V**):										
a. less than 2 min	**0.55**	0.35	0.46*	0.34	0.24	0.02	0.13	−0.07	0.21	−0.06
b. 2 to 20 min	0.27	0.08	0.12	0.13	0.14	0.47*	**0.64**	**0.53**	**0.71**	0.44*
c. 20 min to 1 h	0.17	0.08	−0.03	−0.02	0.13	**0.60**	**0.65**	**0.54**	**0.83**	**0.74**
d. several hours	−0.08	−0.01	−0.09	−0.08	−0.08	**0.63**	**0.52**	**0.59**	**0.71**	**0.68**
e. more than 12 h	−0.02	0.25	−0.02	0.06	−0.03	0.43*	0.06	**0.51**	**0.52**	0.36
02. Heart/chest pain (A***)	**0.52**	**0.52**	**0.67**	**0.52**	**0.52**	−0.06	0.04	0.09	−0.14	−0.19
03. Hot or cold spells (A)	**0.66**	**0.61**	**0.67**	**0.59**	**0.65**	0.16	0.07	0.25	0.16	0.12
04. Falling over (V)	0.40*	0.42*	0.28	0.27	0.40*	0.24	0.20	0.44*	0.36	0.23
05. Nausea, feeling sick (V)	0.47*	**0.50**	**0.58**	0.40*	**0.56**	0.32	0.14	0.35	0.36	0.23
06. Muscle tension/sore (A)	**0.63**	**0.68**	**0.81**	**0.67**	**0.57**	0.08	0.14	0.01	0.01	0.18
07. Light-headed/giddy (V):										
a. less than 2 min	**0.54**	0.28	**0.50**	0.43*	0.28	0.05	0.17	−0.07	0.11	−0.06
b. 2 to 20 min	0.40	0.18	0.30	0.17	0.11	**0.60**	**0.65**	0.46*	**0.70**	**0.53**
c. 20 min to 1 h	0.21	0.08	0.13	0.18	0.08	**0.73**	**0.77**	**0.65**	**0.70**	**0.74**
d. several hours	0.11	0.10	0.08	0.04	0.12	**0.70**	**0.56**	**0.71**	**0.73**	**0.69**
e. more than 12 h	0.05	0.24	0.17	0.02	−0.09	0.45*	0.16	**0.50**	0.46*	**0.55**
08. Trembling, shivering (A)	**0.56**	**0.63**	**0.67**	**0.56**	**0.53**	0.15	0.08	0.27	0.02	0.16
09. Pressure in the ear (A)	0.41*	**0.57**	**0.60**	0.27	0.46*	0.06	0.12	0.16	0.24	0.09
10. Heart pounding (A)	**0.54**	**0.59**	**0.58**	**0.66**	**0.64**	0.10	−0.12	0.23	0.02	0.09
11. Vomiting (V)	0.22	0.15	0.30	0.01	0.26	0.12	0.22	0.28	0.32	0.38
12. Heavy feeling arms/legs (A)	**0.58**	**0.74**	**0.72**	**0.59**	**0.70**	0.24	0.04	0.04	0.04	0.02
13. Visual disturbances (A)	0.42*	**0.71**	**0.72**	**0.58**	**0.61**	0.11	0.14	0.16	0.10	0.18
14. Headache/pressure (A)	**0.57**	0.48*	**0.71**	**0.52**	**0.58**	0.11	0.18	0.23	0.06	0.11
15. Unable to stand/walk (V)	0.16	0.40*	0.38	0.27	**0.54**	0.42*	0.18	0.40*	0.35	0.35
16. Breathing difficulties (A)	**0.63**	**0.69**	**0.62**	**0.53**	**0.72**	0.05	−0.07	0.08	0.06	−0.02
17. Loss of concentration (A)	**0.56**	**0.59**	**0.64**	**0.61**	**0.72**	0.28	0.05	−0.03	0.05	0.15
18. Feeling unsteady (V):										
a. less than 2 min	**0.60**	0.27	**0.54**	0.42	0.24	0.22	0.22	0.13	0.13	−0.04
b. 2 to 20 min	0.39	0.12	0.28	0.13	0.10	**0.64**	**0.64**	**0.62**	**0.71**	**0.58**
c. 20 min to 1 h	0.28	0.02	0.17	0.06	0.13	**0.75**	**0.76**	**0.66**	**0.74**	**0.77**
d. several hours	−0.00	−0.06	−0.12	−0.03	0.08	**0.70**	**0.60**	**0.69**	**0.77**	**0.68**
e. more than 12 h	0.03	0.25	−0.04	0.00	−0.06	**0.51**	0.29	**0.60**	**0.61**	0.49*
19. Tingling, prickling (A)	**0.55**	**0.60**	**0.75**	**0.59**	**0.63**	−0.00	0.05	0.00	−0.02	0.06
20. Pain in the lower back (A)	**0.59**	**0.56**	**0.71**	**0.56**	**0.66**	−0.04	−0.07	−0.08	−0.20	−0.09
21. Excessive sweating (A)	**0.57**	**0.60**	0.19	0.47	**0.60**	0.21	0.05	0.19	0.12	0.18
22. Feeling faint, black out (A)	**0.51**	**0.61**	0.02	0.45	**0.60**	0.19	0.12	0.26	0.30	0.06

The table is adapted from Tschan (Germany, Mainz) [19] and completed with the results of present study Switzerland, (CH) Interdisciplinary Centre for Vertigo & Balance Disorders, University Hospital Zurich. Factor loadings ≥0.5 are highlighted in bold face; * highest factor loading in analysis when ≥0.4 (but ≤0.5). As grey background is not acceptable, it was necessary to add the (V) or (S) definition for ich item; ** (V)= item attributed to VSS-VER; ***(A)= item attributed to VSS-AA.

The VSS-VER subscale showed not such a clearly item matching. Only 47% (9/19) of the items were attributed to the VSS-VER based on their loadings >0.5 on factor two. Four items with loadings from 0.4 to 0.5 (1b, 1e, 7e, 15) were added to VSS-VER because this loading was clearly higher on factor two than factor one. Three items (4, 5, 11) showed unclear loadings either on factor one or two. They were allocated to VSS-VER for the following reason. Item 04 “falling over” showed inconsistent loading across the studies but was attributed to VSS-VER, due to its face validity as a possible symptom in acute vertigo attacks. Similarly, the items 05 “Nausea” and 11 “Vomiting” are two generic symptoms of the autonomous system and showed, as expected, somewhat higher loadings on factor one (VSS-AA). Nevertheless, because of their association to vestibular disorder, particularly acute vertigo attack, these symptoms were attributed to VSS-VER. Another issue was the clear loading on factor one (VSS-AA) of vestibular symptoms lasting less than 2 minutes (items 1a, 7a, 18a). This result seems to confirm a trend observed in other works (Table 2) [5,14,19] and raises the assumption whether this result represents the interface between vertigo and anxiety [4] and thus, could be interpreted as an exacerbation of autonomic arousal leading to vertigo.

Due to the apparently high stability in face of factor extraction, particularly for the VSS-AA subscale and the already documented validity of the structure of the VSS in general [5,14,19], we suggest to keep Yardley’s original subscale structure for our VSS-G.

The cumulative percentage of the scores of the VSS-G, VSS-VER and VSS-AA were analysed. There was no floor effect of the VSS-G and the subscales to be assumed in our sample because the respective scores of the 15 percent threshold [33] lays above the minimal possible score of zero: VSS-G = 16–17, VSS-VER = 6–7, VSS-AA = 5–6. The same holds true for the ceiling effect as the scores of the 85 percent threshold [33] lays clearly under the respective maximal score: VSS-G = 59–60 (maximal score = 136), VSS-VER = 32-33(max = 76), VSS-AA = 30 (max = 60).

#### Reliability determination

All the Cronbach’s α-coefficient estimations showed good internal consistency on scale-level (Table 3). This was also the case on item-level, as there were no differences > 0.1 in Cronbach’s α-coefficient estimation in case the respective item was deleted (Additional file 2) and the CI-TC showed no value under 0.2. For test-retest reliability estimations, the mean time between the first and the second measurement was 5.5 days (SD 1.97 days). As shown in Table 3, all the ICC coefficients reached the recommended threshold of 0.75 [29].

**Table 3:** Internal validity, reliability, discriminant validity results

		**VSS-G**	**VSS-VER**	**VSS-AA**
**Cronbach's α [n = 202]**		.904	.859	.864
**ICC (CI) [n = 40]**		.926 (.826/.965)	.920 (.854/.957)	.913 (.737/.963)
**Medians Healthy [n = 52] vs. peripheral vest disorder [n = 90]**	**U**	326.50	43.00	1046.50
	**Z**	−8.529*	−9.782*	−5.486 8*
**Medians Healthy [n = 52] vs. central vest. disorder [n = 73]**	**U**	153.500	22.000	467.500
	**Z**	−8.742*	−9.467*	−7.171*
**Medians Healthy [n = 52] vs. multifactorial disorder [n = 39]**	**U**	89.50	62.00	210.00
	**Z**	−7.421*	−7.797*	−6.456*
**No anxiety [n=166] vs. abnormal anxiety [n = 36]**	**U**	1776.50	2446.00	1427.00
	**Z**	−3.811*	−1.706	−4.912*

* Significant (2 tailed) at a <0.001 level.

VSS-G = VSS total score, VSS-VER = VSS vertigo subscale, VSS-AA = VSS somatic anxiety subscale. Mann–Whitney-*U*-test (U) 1) for comparison of median VSS-VER scores between healthy and diagnostic groups, 2) median VSS-AA scores between patients without and patients with anxiety (HADS-A ≥11).

#### External validity determination

##### Discriminant validity

**Figure 2:**
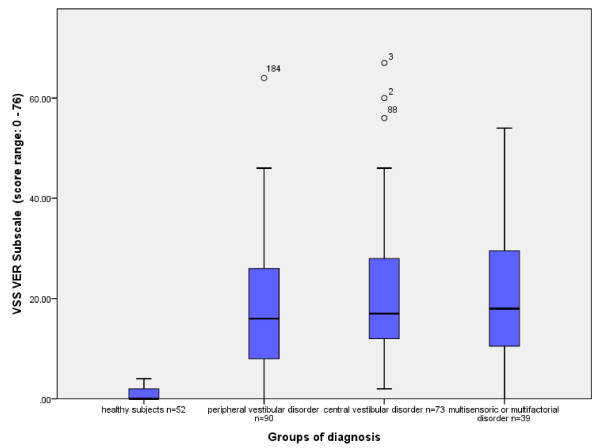
Medians and interquartile range of VSS-VER subscores of the three diagnostic groups and healthy subjects.

**Figure 3:**
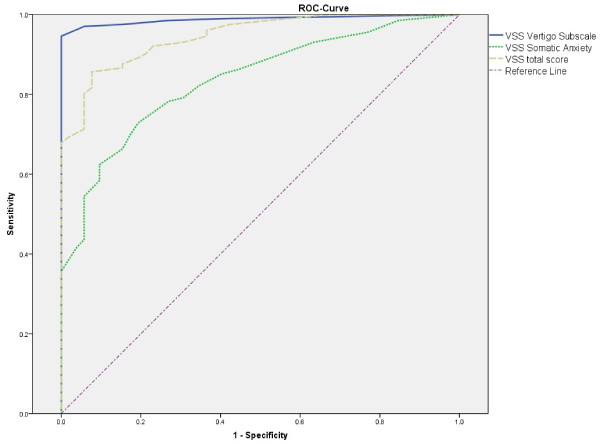
ROC-Curve for discrimination between dizzy patients and healthy subjects.

The Mann–Whitney-*U*-Test showed significant differences in the distribution of the median scores of the VSS-G, the VSS-VER (Figure 2) and VSS-AA between healthy subjects and persons with vestibular disorders (Table 3). Therefore, the areas under the ROC-Curve (CI) were calculated: VSS-G = 0.95 (0.92, 0.97), VSS-VER = 0.99 (0.98, 1.00) and VSS-AA = 0.84 (0.78, 0.89) (Figure 3). According to the Youden Index [31] the best relationship of 0.95 sensitivity and 1.00 specificity could be set at VSS-VER Score = 4.5 (or 0.24 item mean). Particularly the VSS-VER (Figure 2) was able to discriminate between healthy subjects and persons with vestibular disorders. But there was no skill to differentiate between the groups of diagnosis. The distribution of median scores of VSS-AA showed a significant difference between people without and those with abnormal anxiety as defined by the HADS-A (Score ≥ 11). However, the ROC-Curve showed very low specificity.

##### Convergent validity

**Figure 4:**
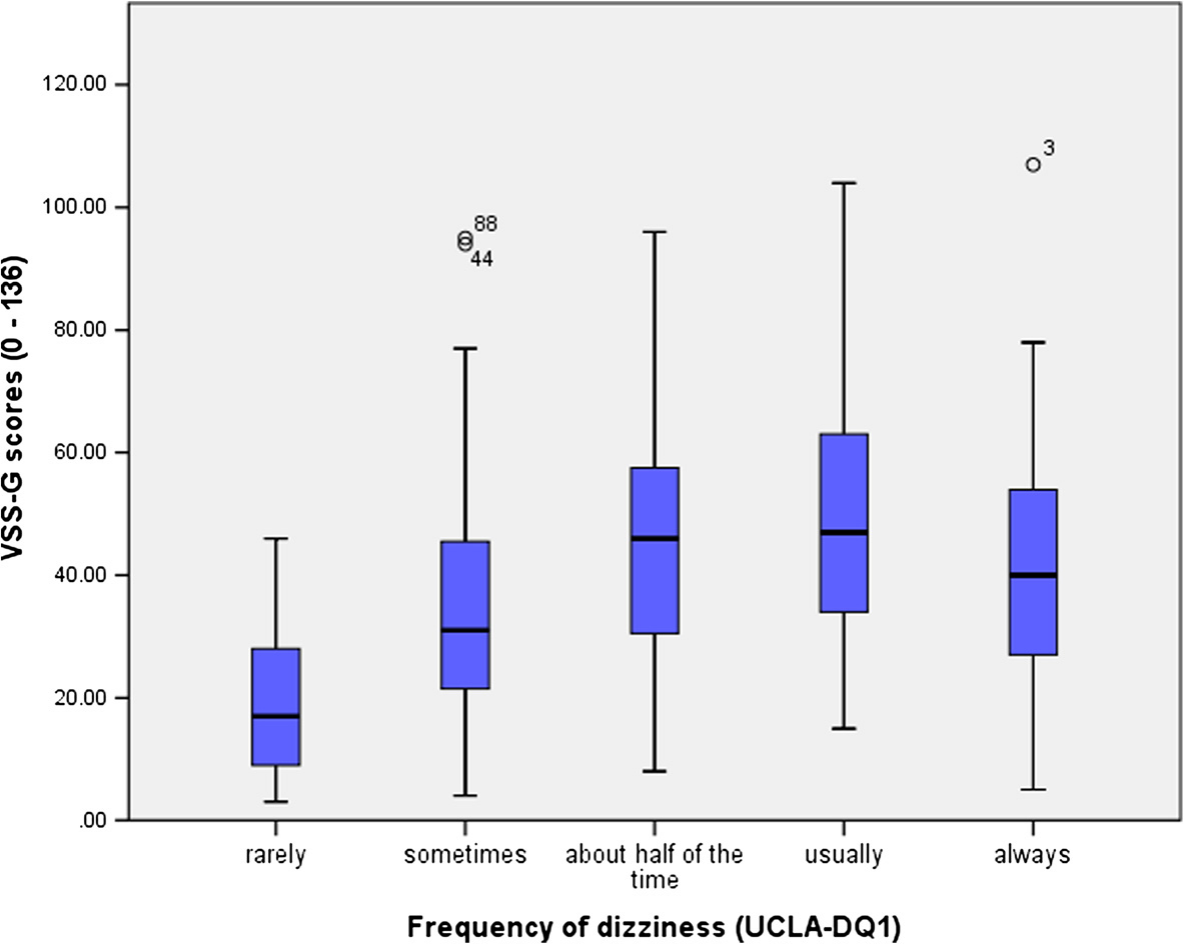
VSS-G median scores plotted to dizziness frequency (UCLA-DQ1).

In order to confirm the structure of the subscale regarding the possible interaction with anxiety, a different correlation of VSS-VER and VSS-AA with measurement of anxiety (HADS-A) was expected. According to the definition of the thresholds of Gill-Body [34], the VSS-AA correlated fairly with the HADS-A, while there was only low correlation between the VSS-VER and the HADS-A (Table 4). Furthermore, the VSS-G showed moderate correlation with self-perceived disability as measured by the DHI (Table 4). The Kendall Tau rank correlation coefficient (Table 5) showed a significant but only fair correlation between the VSS-G and the self-rated frequency of dizziness (UCLA-DQ1), this correlation could partly be displayed by the box plot of the VSS-G median scores against the UCLA-DQ1 (Figure 4). The VSS-G total score and both subscores correlated fairly with self-estimated (mild, moderate, severe) disability. The impact on daily activities correlated fairly with VSS-G and VSS-VER; however, it only correlated weakly with VSS-AA.

**Table 4:** Spearman’s correlation coefficients

**Spearman's Rho n = 202**	**VSS-G**	**VSS-VER**	**VSS-AA**	**HADS-A**
VSS-VER	.875^**^			
VSS-AA	.865^**^	.549^**^		
HADS-A	.369^**^	.186^**^	.452^**^	
DHI	.554^**^	.496^**^	.464^**^	.448^**^

**. The correlation is significant on a 0,01 Level (two sided). VSS-G = VSS total score, VSS-VER = VSS vertigo subscale, VSS-AA = VSS somatic anxiety subscale, DHI = Dizziness Handicap Inventory total score, HADS-A = Hospital Anxiety and Depression Scale - Anxiety subscale).

**Table 5:** Kendall’s Tau rank correlation coefficients of VSS with self-estimated disability and 3 items of UCLA-DQ

**Kendall-Tau n = 202**	**VSS-G**	**VSS-VER**	**VSS-AA**	**frequency**	**intensity**	**disability**
VSS-VER	.711^**^					
VSS-AA	.703^**^	.397^**^				
frequency (UCLA-DQ1)	.317^**^	.262^**^	.299^**^			
intensity (UCLA-DQ2)	.227^**^	.275^**^	.108^*^	.101		
disability	.400^**^	.385^**^	.321^**^	.369^**^	.469^**^	
impact on daily activities (UCLA-DQ3)	.281^**^	.309^**^	.172^**^	.133^*^	.590^**^	.508^**^

** correlation is significant on a 0,01 Level (two sided).

* correlation is significant on a 0,05 Level (two sided).

VSS-G = VSS-G total score, VSS-VER = VSS vertigo subscale, VSS-AA = VSS somatic anxiety subscale; UCLA-DQ1 = self-estimated frequency of dizziness, UCLA-DQ2 = self-estimated intensity of dizziness, UCLA-DQ3 = self-estimated impact of dizziness on daily activities; disability = self-rated mild, moderate, severe disability.

## Discussion

The VSS was translated and cross-culturally adapted into German for a wider German speaking population of Switzerland. The original’s structure of the subscales could be confirmed and acceptable psychometric properties were found. The VSS-G may be adopted to collect the self-perceived symptoms of patients with vestibular disorders and to integrate the information in the treatment plan.

### Internal validity determination

#### Reliability determination

The VSS-G translation exhibits high internal consistency and acceptable test-retest reliability, similar to the original VSS and its subscales [14]. This indicates the ability of the translated instrument to reliably screen groups of patients. The aspect of agreement was not analysed in the study [35]; however, as the VSS was conceived to screen the frequency of the symptoms rather than to measure change, this was not deemed a compulsory psychometric characteristic. However, we admit that measuring change remains an important topic in vestibular rehabilitation. The short form of the VSS [36,37], for which acceptable limits of agreement were found in a Turkish translation [38], seems to be an appropriate instrument for this purpose. The findings of this study justify a German language translation for the short form VSS for the purpose of establishing an instrument capable of measuring change in symptoms.

#### External validity determination

##### Discriminant validity

Particularly the VSS-VER subscale was able to discriminate between healthy subjects and people with dizziness. If the determined VSS-VER cut-off score would be applied, eleven participants out of 252 (4.5%) were misclassified as false negatives. This may indicate that the vestibular symptoms of these patients are very low, which may mislead to scrutinise the effectiveness of a vestibular rehabilitation. Therefore, particularly for these patients all the aspects of the health problems like comorbidities and limitations in activities of daily life and participation should be considered in the treatment plan. Anyway, further investigations were necessary to analyse the usefulness of this cut-off score prior to vestibular rehabilitation. The low discriminant properties for vestibular disorders of the VSS-AA may highlight the interface between vestibular dysfunction and disorders of autonomous system and anxiety. In contrast the VSS-AA subscale seemed capable to screen for possible abnormal anxiety, but it seems fair to state that further research is needed on this discriminative property.

##### Convergent validity

The low association of the VSS-VER with the HADS-A seems to imply that our finding might confirm Yardley’s research for a tool to measure the severity of vertigo symptoms, which is supposed to be uncontaminated by anxiety [14]. On the other hand, the VSS-AA’s fair association with HADS-A and its discriminant property for abnormal anxiety, may suggest that the symptoms of VSS-AA might be associated with an anxiety disorder. This may confer to the subscale a certain ability to screen for possible anxiety problems without providing any other conclusive information about anxiety (e.g. aetiology of comorbidity). This property may be helpful in rehabilitation management to counsel the patients and refer them to health professionals experienced in psychological disorders for further assessment.

The influence of the severity of the symptoms on limitations of daily activities was showed by correlations of the VSS-G with measurements of dizziness related disability in daily activities (DHI, UCLA-DQ3) and the self-estimated disability due to dizziness. These findings are comparable to the work of Tamber et al.[39], who showed an association between the Norwegian VSS short-form and the DHI; and Yardley who measured disability with the Vertigo Handicap Questionnaire [5,14].

Further than disability, symptoms may also have some influence on health-related quality of life (HRQoL). This is an important issue, as HRQoL measurement figures out the dimension of the impact of dizziness on the patient’s participation to social life. 1) In order to measure the outcome of vestibular rehabilitation Morris et al. [40,41] developed the Vestibular Rehabilitation Benefit Questionnaire (VRBQ) with a HRQoL subscale, which showed a moderate correlation to the VSS. However, this result should be interpreted cautiously, because there were VSS items included in this VRBQ subscale. Nevertheless the scale seems useful for measuring changes in vestibular rehabilitation. 2) Yanik [38] translated and validated the VSS short-form and the Vertigo, Dizziness and Imbalance questionnaire (VDI) [42] (containing a HRQoL subscale) into Turkish, unfortunately there were no intercorrelations reported. 3) In her work, Tschan[19] analysed the correlations of the VSS with generic quality of life measurement (physical and mental health components of the SF-36). She found remarkably weaker correlations with HRQoL for the VSS-VER subscale than for the VSS-AA [19]. However, these results should be interpreted cautiously because of a possible lack of relationship between the SF-36 measure and the salient areas of dizziness. Previously, Prieto et al. [42] recommended the VDI for more relevant and responsive measurements of health-related quality of life. However, current in-depth research on this instrument is lacking, which impedes the possible discussion about which instrument should be preferred in future trials. Nevertheless, the findings of Tschan underpin the influence of emotional or psychological distress on generally perceived quality of life, which should also be considered in vestibular rehabilitation.

As both scales measure frequency, it may be surprising that the plot of the VSS-G median scores to the frequency of dizziness (UCLA-DQ1, Figure 4) is not more distinct. The mismatch seems to appear with increasing frequency (Figure 4) and is probably due to the different structure of the instruments. As a matter of fact, in the VSS-G several item scores were added, while the UCLA-DQ1 is a single overall estimation of the frequency of dizziness. Possibly there are some confounders influencing frequency: 1) the described interaction of intensity [43] and/or 2) the non-negligible mutual influence between the symptoms of both subgroups (VSS-VER and VSS-AA). Further research is needed to illustrate the impact of all these possible interactions.

Further research is also needed to determine the extent of usefulness of VSS in vestibular rehabilitation such as the determination of cut-off scores which produce a relevant predictive evidence for rehabilitation success on the individual patient level.

#### Limitations

The present study was not without limitations; the capacity of the VSS-VER subscale to differentiate between the designed diagnostic groups could not be reproduced like in other similar studies. Our group allocation hampered comparison of the results unlike those in other studies with somewhat other classifications. However, it showed to be appropriate when targeting vestibular rehabilitation. Furthermore, this group allocation yielded quite unequal group sizes which, in turn, could have biased the results. Therefore, calculations were carried out with random selections of cases within the groups in order to balance the group sizes, and the described differences were maintained. The fact that in the meantime a German translation of the VSS was published [19] could be seen as a further limitation. However, although this translation by a German team was published during our data collection process, too late to influence the generation of our version, our work cannot be outclassed for several reasons. Firstly, our study methodology [16,17] took in consideration cultural and language differences between the German-speaking population of Switzerland [18]. Secondly, the broad group allocation in peripheral, central and multifactorial vestibular disorders was designed to validate the VSS-G for screening people with vertigo, dizziness or balance disorders prior to vestibular rehabilitation. Thirdly, the convergent validity analysis carried out by Tschan and colleagues did not use any dizziness-specific measurement of disability.

## Conclusions

The present German translation of the VSS shows satisfactory psychometric properties for the assessment of the self-perceived severity of the symptoms on a patient group level in a large geographic area of German-speaking countries. The high discriminant validity of the VSS-VER subscale allows it to be used for screening vestibular dysfunction and coordinate the treatment. The VSS-AA subscale is able to screen for symptoms which might refer to an anxiety disorder, which might be addressed additionally in the context of vestibular rehabilitation.

## Competing interests

The authors declare that there are no competing interests.

## Authors' contributions

TG-J contributed to the design of the survey. He conducted the statistical analysis and wrote the manuscript. AK co-initiated the study and contributed to the design of the study and to the process of data collection and interpretation. She reassessed the article. DS contributed to the analysis of data and revised the article thoroughly for its content. As the co-director of the interdisciplinary centre for vertigo and balance disorders, he bore the medical responsibility of the study. EDdB contributed to the interpretation of the data and revised the article critically for its content. All authors read and approved the final manuscript.

## Pre-publication history

The pre-publication history for this paper can be accessed here:

http://www.biomedcentral.com/1472-6815/12/7/prepub

## Supplementary Material

Additional file 1 Final validated VSS-G version. Click here for file

Additional file 2 Reliability results on item level. Click here for file
