# Supplementary material for: Translation and validation of the vertigo symptom scale into German: A cultural adaption to a wider German-speaking population
Source: BMC Ear Nose Throat Disord. 2012 Jul 2;12:7. doi: 10.1186/1472-6815-12-7 (PMC3441555; doi:10.1186/1472-6815-12-7)
Supplement: Additional file 1 — Final validated VSS-G version. [file 1472-6815-12-7-S1.doc]

|  | **ICC Item**  **(CI)** | **VSS-G** | | **VER** | | **AA** | |
| --- | --- | --- | --- | --- | --- | --- | --- |
|  |  | CI-TC | α item deleted | CI-TC | α item deleted | CI-TC | α item deleted |
| 1. Feeling Things spinning / Moving: |  |  |  |  |  |  |  |
| a. less than 2 min | .868 (.766/ .928) | .400 | .902 | .331 | .859 |  |  |
| b. 2 to 20 min | .753 (.581/ .861) | .434 | .902 | .505 | .851 |  |  |
| c. 20 min to 1h | .728 (.544/ .846) | .440 | .902 | .535 | .850 |  |  |
| d. several hours | .702 (.500/ .830) | .291 | .903 | .390 | .855 |  |  |
| e. more than 12h | .672 (.459/ .812) | .242 | .904 | .287 | .858 |  |  |
| 2. Heart/ chest pain | .680 (.471/ .817) | .352 | .903 |  |  | .484 | .857 |
| 3. Hot or cold spells | .624 (.353/ .790) | .578 | .899 |  |  | .624 | .849 |
| 4. Falling over | .725 (.537/ .844) | .420 | .902 | .362 | .856 |  |  |
| 5. Nausea, feeling sick | .762 (.578/ .869) | .529 | .900 | .511 | .850 |  |  |
| 6. Muscle tension / sore | .870 (.769/ .929) | .514 | .900 |  |  | .586 | .851 |
| 7. Light-headed / giddy: |  |  |  |  |  |  |  |
| a. less than 2 min | .758 (.588/ .864) | .398 | .902 | .355 | .858 |  |  |
| b. 2 to 20 min | .733 (.549/ .850) | .608 | .899 | .671 | .843 |  |  |
| c. 20 min to 1h | .745 (.568/ .856) | .536 | .900 | .625 | .845 |  |  |
| d. several hours | .798 (.651/ .888) | .472 | .901 | .514 | .850 |  |  |
| e. more than 12h | .748 (.573/ .858) | .291 | .904 | .272 | .861 |  |  |
| 8. Trembling, shivering | .522 (.253/ .716) | .493 | .901 |  |  | .559 | .853 |
| 9. Pressure in the ear | .865 (.742/.929 ) | .330 | .904 |  |  | .379 | .863 |
| 10. Heart pounding | .904 (.796/ .952) | .444 | .901 |  |  | .491 | .856 |
| 11. Vomiting | .722 (.533/ .842) | .220 | .904 | .222 | .860 |  |  |
| 12. Heavy feeling arms / legs | .823 (.680/ .904) | .570 | .899 |  |  | .580 | .851 |
| 13. Visual disturbances | .762 (.578/ .869) | .359 | .903 |  |  | .368 | .862 |
| 14. Headache / pressure in the head | .912 (.803/ .957) | .483 | .901 |  |  | .558 | .853 |
| 15. Unable to stand / walk | .521 (.253/ .715) | .336 | .903 | .402 | .855 |  |  |
| 16. Breathing difficulties | .903 (.820/ .948) | .480 | .901 |  |  | .551 | .854 |
| 17. Loss of concentration | .871 (.765/ .930) | .559 | .899 |  |  | .537 | .854 |
| 18. Feeling unsteady: |  |  |  |  |  |  |  |
| a. less than 2 min | .775 (.614/ .874) | .545 | .900 | .478 | .852 |  |  |
| b. 2 to 20 min | .778 (.618/ .876) | .622 | .899 | .675 | .843 |  |  |
| c. 20 min to 1h | .877 (.781/ .933) | .615 | .899 | .723 | .842 |  |  |
| d. several hours | .722 (.531/ .843) | .380 | .902 | .479 | .852 |  |  |
| e. more than 12h | .837 (.715/ .910) | .308 | .903 | .369 | .856 |  |  |
| 19. Tingling, prickling | .887 (.797/ .939) | .415 | .902 |  |  | .543 | .854 |
| 20. Pain in the lower back | .876 (.779/ .932) | .397 | .902 |  |  | .500 | .856 |
| 21. Excessive sweating | .915 (.846/ .954) | .516 | .900 |  |  | .476 | .857 |
| 22. Feeling faint, about to black out | .925 (.860/ .960) | .464 | .901 |  |  | .402 | .860 |

ICC: Intra-class correlation; CI-TC: corrected item total correlation; α item deleted: Cronbach’s α correlation coefficient with the respective item deleted.
