# Supplementary material for: Translation and validation of the vertigo symptom scale into German: A cultural adaption to a wider German-speaking population
Source: BMC Ear Nose Throat Disord. 2012 Jul 2;12:7. doi: 10.1186/1472-6815-12-7 (PMC3441555; doi:10.1186/1472-6815-12-7)
Supplement: Additional file 2 — Reliability results on item level. [file 1472-6815-12-7-S2.pdf]

VERTIGO SYMPTOM SCALE (VSS-G)
SYMPTOME BEI SCHWINDEL

Anleitung:
Bitte kreuzen Sie die entsprechende Spalte an, um damit anzugeben, wie oft jedes der unten aufgeführten Symptome während der letzten 12 Monate aufgetreten ist. Falls Sie seit weniger als einem Jahr unter Schwindel leiden, geben Sie bitte an, wie oft jedes der unten aufgeführten Symptome seither aufgetreten ist.

Mögliche Antworten sind:

nie	wenige Male = 1-3 Mal im Jahr	mehrere Male = 4-12 Mal im Jahr	ziemlich oft = durchschnittlich mehr als einmal im Monat	sehr oft = durchschnittlich mehr als einmal die Woche

Wie oft traten bei Ihnen **in den letzten 12 Monaten** folgende Symptome auf?

	nie	wenige Male	mehrere Male	ziemlich oft	sehr oft
1a. Ein Gefühl, dass sich Dinge bewegen oder drehen, welches **weniger als 2 Minuten** dauerte	❐	❐	❐	❐	❐
1b. Ein Gefühl, dass sich Dinge bewegen oder drehen, welches **bis zu 20 Minuten** dauerte	❐	❐	❐	❐	❐
1c. Ein Gefühl, dass sich Dinge bewegen oder drehen, welches **20 Minuten bis zu 1 Stunde** dauerte	❐	❐	❐	❐	❐
1d. Ein Gefühl, dass sich Dinge bewegen oder drehen, welches **mehrere Stunden** dauerte	❐	❐	❐	❐	❐
1e. Ein Gefühl, dass sich Dinge bewegen oder drehen, welches **mehr als 12 Stunden** dauerte	❐	❐	❐	❐	❐
2. Schmerzen in der Herz- oder Brustgegend	❐	❐	❐	❐	❐
3. Vorübergehende Hitze- oder Kältegefühle	❐	❐	❐	❐	❐
4. Ein so starkes Schwanken, dass Sie wirklich stürzten	❐	❐	❐	❐	❐

	**nie**	**wenige Male**	**mehrer e Male**	**ziemlich oft**	**sehr oft**
5. Übelkeit; ein Gefühl, dass sich der Magen umdreht	❐	❐	❐	❐	❐
6. Verspannung / Schmerzhaftigkeit der Muskeln	❐	❐	❐	❐	❐
7a. Sich benommen, schwebend oder schwindlig zu fühlen. Dieses Gefühl dauerte **weniger als 2 Minuten**	❐	❐	❐	❐	❐
7b. Sich benommen, schwebend oder schwindlig zu fühlen. Dieses Gefühl dauerte **bis zu 20 Minuten**	❐	❐	❐	❐	❐
7c. Sich benommen, schwebend oder schwindlig zu fühlen. Dieses Gefühl dauerte **20 Minuten bis zu 1 Stunde**	❐	❐	❐	❐	❐
7d. Sich benommen, schwebend oder schwindlig zu fühlen. Dieses Gefühl dauerte **mehrere Stunden**	❐	❐	❐	❐	❐
7e. Sich benommen, schwebend oder schwindlig zu fühlen. Dieses Gefühl dauerte **mehr als 12 Stunden**	❐	❐	❐	❐	❐
8. Zittern, Frösteln, Schütteln	❐	❐	❐	❐	❐
9. Druckgefühl in einem Ohr oder beiden Ohren	❐	❐	❐	❐	❐
10. Herzklopfen, unregelmässiger Herzschlag oder Herzrasen	❐	❐	❐	❐	❐
11. Erbrechen	❐	❐	❐	❐	❐
12. Gefühl von Schwere in Armen oder Beinen	❐	❐	❐	❐	❐
13. Sehstörungen (z.B. verschwommenes Sehen, Flimmern, Punkte vor den Augen)	❐	❐	❐	❐	❐
14. Kopfschmerzen oder Druckgefühl im Kopf	❐	❐	❐	❐	❐

	nie	wenige Male	mehrere Male	ziemlich oft	sehr oft
15. Unfähigkeit ohne Unterstützung richtig zu stehen oder zu gehen	❐	❐	❐	❐	❐
16. Atemschwierigkeiten, Kurzatmigkeit	❐	❐	❐	❐	❐
17. Konzentrations- oder Gedächtnisstörungen	❐	❐	❐	❐	❐
18a. Sich unsicher auf den Beinen fühlen; nahe daran sein, das Gleichgewicht zu verlieren. Dieses Gefühl dauerte **weniger als 2 Minuten**	❐	❐	❐	❐	❐
18b. Sich unsicher auf den Beinen fühlen; nahe daran sein, das Gleichgewicht zu verlieren. Dieses Gefühl dauerte **bis zu 20 Minuten**	❐	❐	❐	❐	❐
18c. Sich unsicher auf den Beinen fühlen; nahe daran sein, das Gleichgewicht zu verlieren. Dieses Gefühl dauerte **20 Minuten bis zu 1 Stunde**	❐	❐	❐	❐	❐
18d. Sich unsicher auf den Beinen fühlen; nahe daran sein, das Gleichgewicht zu verlieren. Dieses Gefühl dauerte **mehrere Stunden**	❐	❐	❐	❐	❐
18e. Sich unsicher auf den Beinen fühlen; nahe daran sein, das Gleichgewicht zu verlieren. Dieses Gefühl dauerte **mehr als 12 Stunden**	❐	❐	❐	❐	❐
19. Kribbeln, Prickeln oder Taubheitsgefühl in einzelnen Körperteilen	❐	❐	❐	❐	❐
20. Schmerzen im unteren Teil des Rückens	❐	❐	❐	❐	❐
21. Übermässiges Schwitzen	❐	❐	❐	❐	❐
22. Schwarz werden vor den Augen; kurz davor, ohnmächtig zu werden	❐	❐	❐	❐	❐
